# Supplementary material for: Unusual multiscale mechanics of biomimetic nanoparticle hydrogels
Source: Nat Commun. 2018 Jan 12;9:181. doi: 10.1038/s41467-017-02579-w (PMC5766503; doi:10.1038/s41467-017-02579-w)
Supplement: Supplementary file 1 — Supplementary Information [file 41467_2017_2579_MOESM1_ESM.pdf]

Supplementary Table 1. Nanoparticle parameters for Hydrogel-544, Hydrogel-590, Hydrogel-618 and Hydrogel-657

	Hydrogel-544	Hydrogel-590	Hydrogel-618	Hydrogel-657
Size (nm)	~ 2.7	~ 3.2	~ 3.7	~ 4.0
Molecular Weight (KDa)	~ 38.4	~ 63.3	~ 105.0	~ 123.7

Supplementary Table 2. Nanoparticle parameters for CYS-CdTe, MPA-CdTe and GSH-Au

	CYS-CdTe		MPA-CdTe		GSH-Au	
Size (nm)	~ 3.2	~ 3.7	~ 2.7	~ 3.1	~ 3.0	~ 8.0
Molecular Weight (KDa)	~ 63.3	~ 105.0	~ 38.4	~ 59.9	~ 86.0	~ 3590

Supplementary Table 3. VFOM of NP-based Hydrogels

	Hydrogel-544<br>10 Hz		Hydrogel-590<br>10 Hz		Hydrogel-618<br>10 Hz	
tanδ	~ 0.43		~ 0.51		~ 0.71	
VFOM (MPa)	~ 1.83		~ 1.71		~ 0.21	
	GSH-Au<br>10 Hz		CYS-CdTe<br>10 Hz		MPA-CdTe<br>10 Hz	
	3.0 nm	8.0 nm	3.2 nm	3.7 nm	2.7 nm	3.1 nm
tanδ	~0.34	~0.11	~ 0.19	~ 0.22	~ 0.14	~ 0.18
VFOM (MPa)	~ 0.21	~ 0.005	~ 0.10	~ 0.67	~ 0.06	~ 0.16

Note：1 Hz = 6.283 rad/s

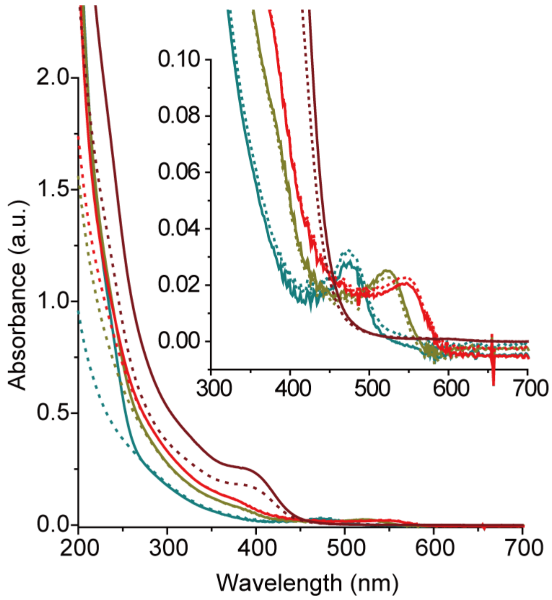

Supplementary Figure 1. UV-vis spectra of GSH-CdTe nanoparticles. The re-dispersed CdTe NPs (dash line) and viscous liquid dispersion (solid line) were obtained after phase separation with isopropanol. (cyan line, NPs-523; dark yellow line, NP-566; red line, NP-600; wine line, NP-647)

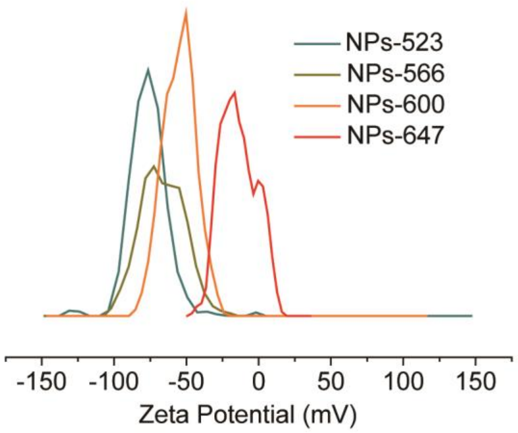

Supplementary Figure 2. Zeta potential measurement of the diluted solution of hydrogels
To test the effective charge for CdTe NPs in Hydrogel-544, Hydrogel-590, Hydrogel-618 and Hydrogel-657, 3 ml fresh solution of CdTe NPs was mixed with 6 ml isopropanol, and then centrifuged at 8000 rpm/min for 3 min. After removing the supernatant, the viscous liquid of CdTe NPs was dissolved in 1 ml DI water for Zeta potential test. As indicated in Supplementary Figure 2, the zeta potential of NPs corresponding to the three hydrogels is -78 mV for NPs-523, -67 mV for NPs-566, -50 mV for NPs-600 and -17 mV for NPs-647, respectively. Zeta potentials corresponding to highly negatively charged NPs indicate good stability in aqueous solution.

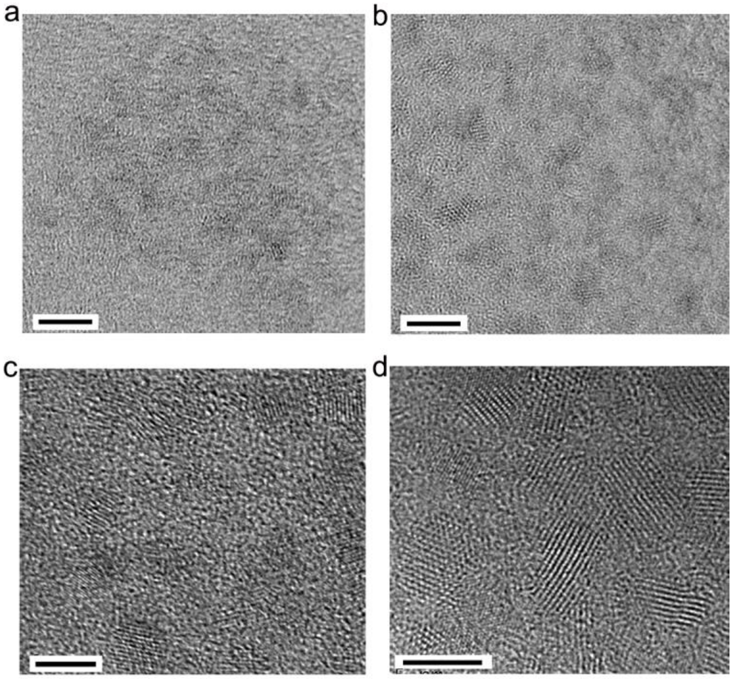

Supplementary Figure 3. High resolution TEM of NPs-523, NPs-566, NPs-600 and NPs-647. Scale bar: 5 nm.

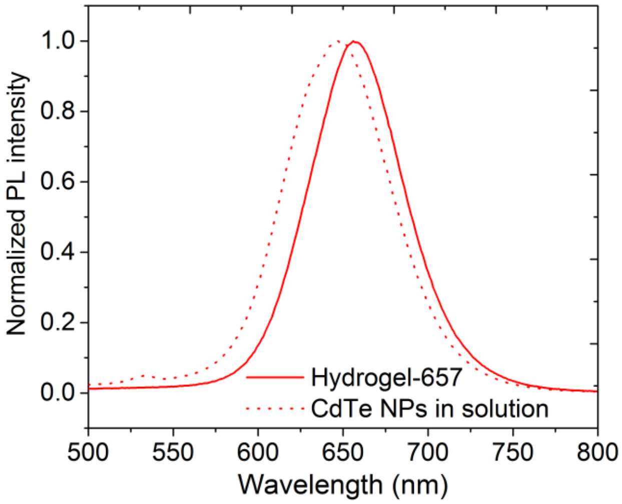

Supplementary Figure 4. Photoluminescence spectra of CdTe NP (~ 4 nm) dispersion (emission peak, 647 nm) and hydrogels (emission peak, 657 nm).

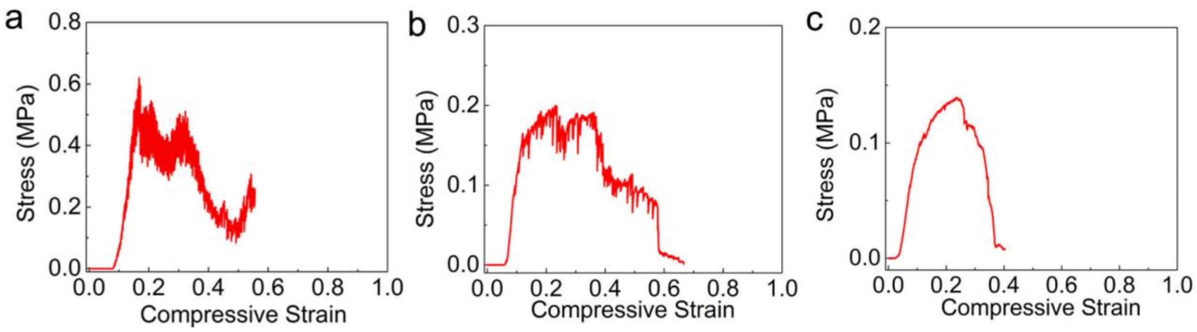

Supplementary Figure 5. Compressive strength of aerogels prepared from (a) Hydrogel-544, (b) Hydrogel-590 and (c) Hydrogel-618. Static compressive testing on cylindrical specimens was conducted on an electronic universal test machine (Sun, UTM2012). All specimen have a diameter of 1.0 cm and a length of 2.0 cm.

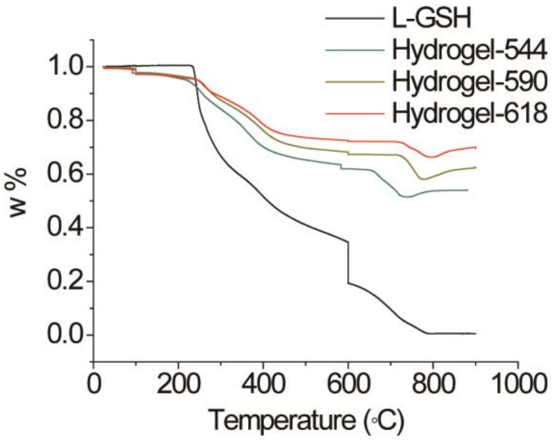

Supplementary Figure 6. Weight loss from thermogravimetric analysis for L-GSH. Detailed discussion for volumetric ratio of the soft GSH shell vs hard CdTe core in Methods.

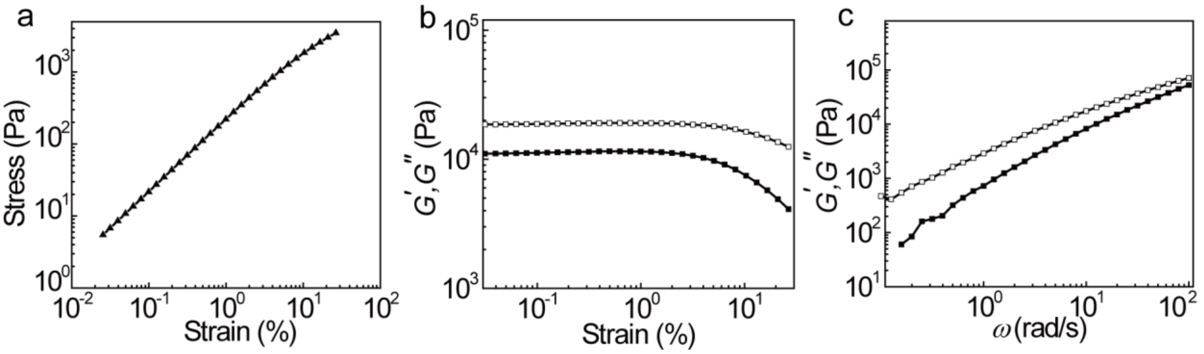

Supplementary Figure 7. Viscoelastic properties of Hydrogel-657. Oscillatory stress/strain (**a**), continuous step moduli/strain (**b**) and rheological dynamic oscillatory frequency sweep (**c**) of GSH-CdTe NP Hydrogel-657. Hydrogels-657 shows lower shear modulus, lower storage moduli and loss moduli than Hydrogels-618. (In Supplementary Figure **7**b, c, solid symbol corresponds to the storage moduli G′ and empty symbol corresponds to the loss moduli G".

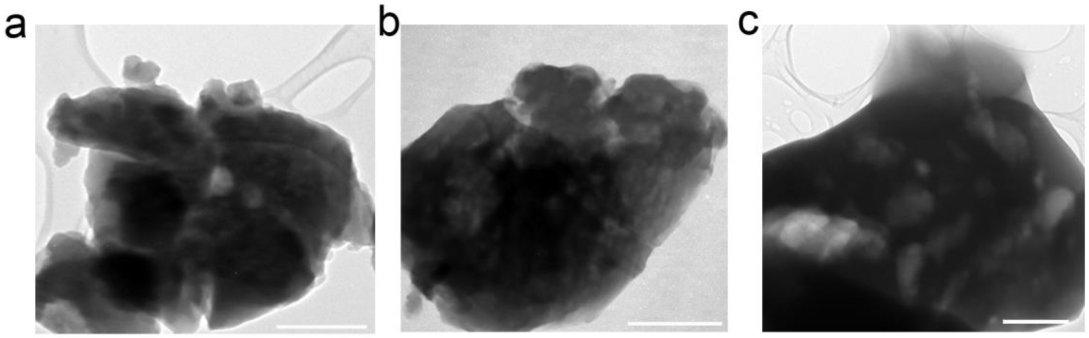

Supplementary Figure 8. TEM characterization of hydrogel-544, Hydrogel-590 and Hydrogel-618. TEM images of the porous solid samples corresponding to Hydrogel-544 (a), Hydrogel-590 (b) and Hydrogel-618 (c). Scale bar: 500 nm.

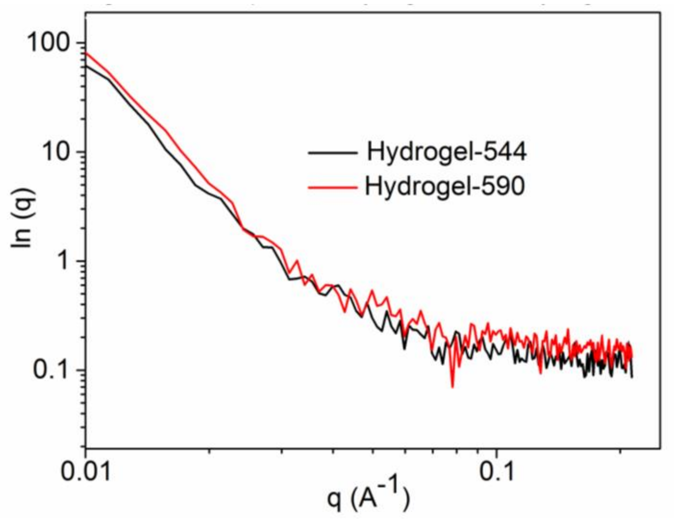

Supplementary Figure 9. Small-angle x-ray scattering (SAXS) measurements. SAXS results show zero-characteristic peaks for Hydrogel-544 and Hydrogel-590, indicating that Hydrogels have similar random structures.

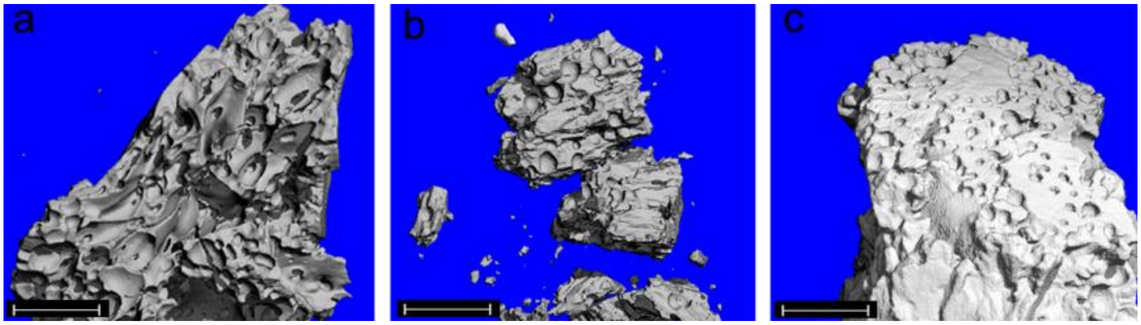

Supplementary Figure 10. X-ray tomography of Hydrogel-544, Hydrogel-590 and Hydrogel-618. All three of hydrogels show highly similar morphology in microscale. Scale bar: 1 mm. (**a**) Hydrogel-544, (**b**) Hydrogel-590 and (**c**) Hydrogel-618.

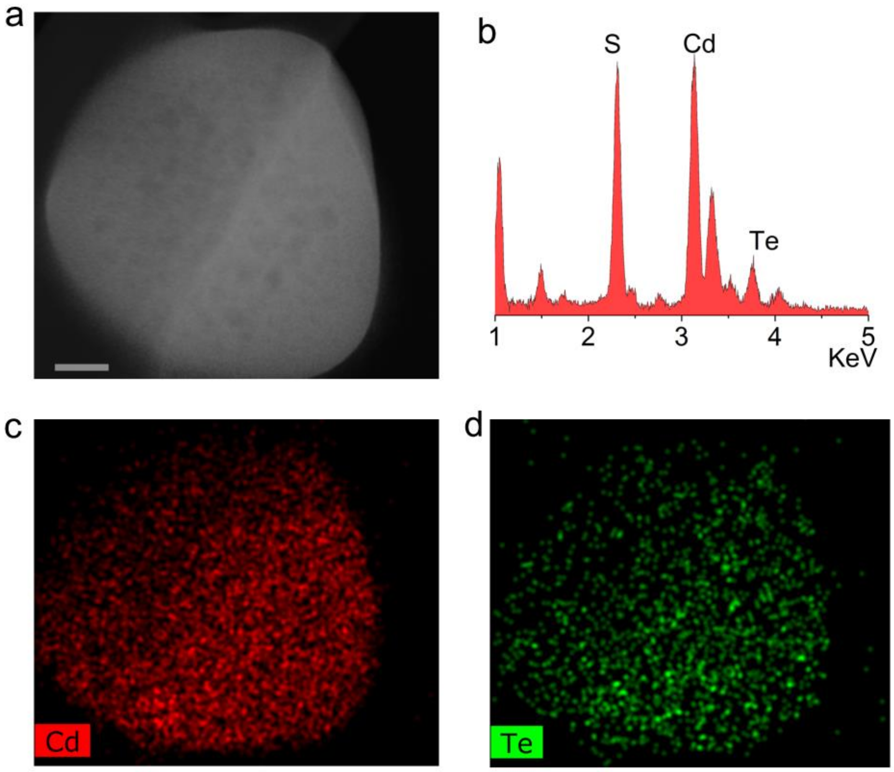

Supplementary Figure 11. Energy-dispersive X-ray elemental mapping of Hydrogel-590. Scale bar in Supplementary Figure 11a is 50 nm.

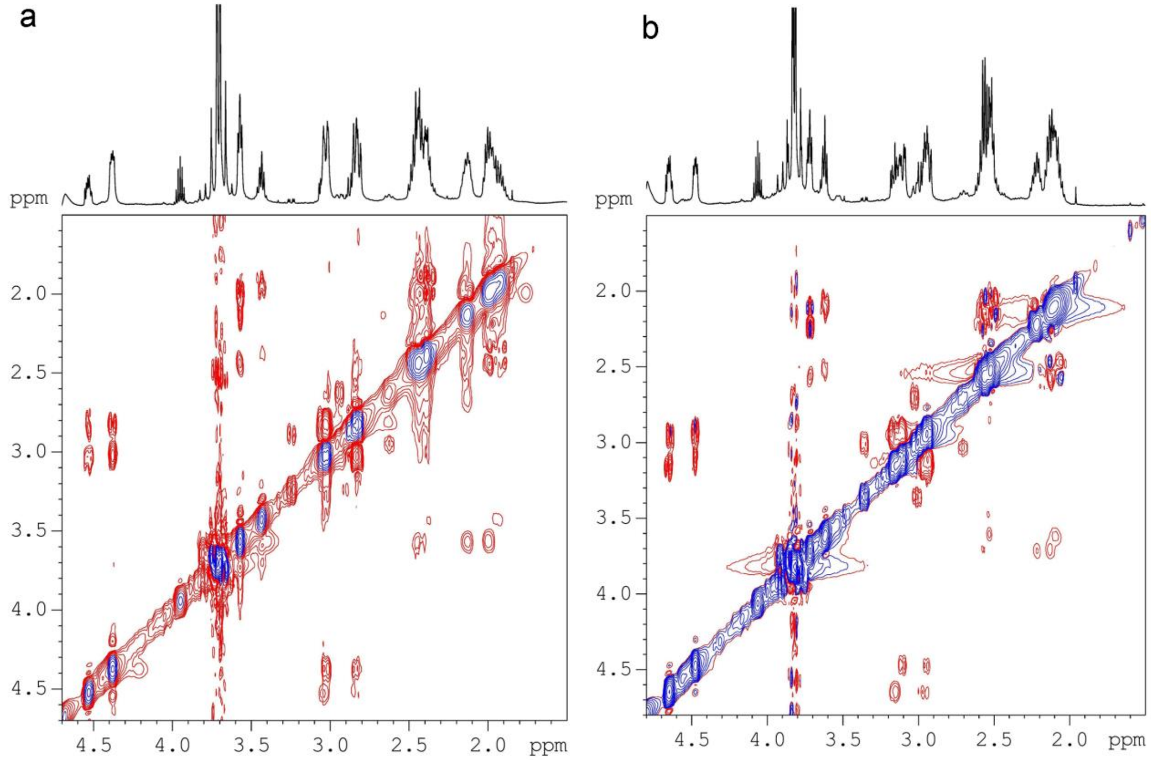

Supplementary Figure 12. Two-dimensional ^{1}H-^{1}H ROESY spectra of Hydrogel-590 and Hydrogel-618. The ^{1}H-^{1}H ROESY spectra of Hydrogel-590 and Hydrogel-618 did not show obvious ligand exchange cross peaks between Cys α and Cys α, and Glu α and Glu α of two major configurations. It is possibly due to the higher molecular weight of the samples compared to Hydrogel-544. As is well known, the intensity of cross peaks depends on molecular correlation time, which is mainly determined by the molecular weight of the sample.

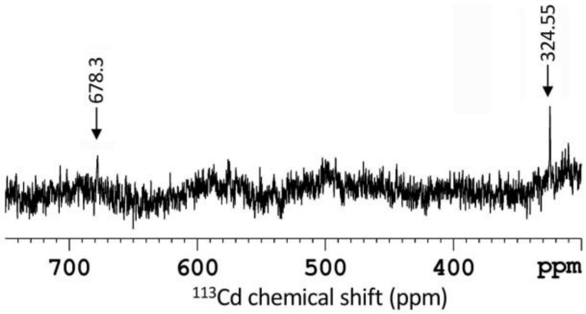

Supplementary Figure 13. One-dimensional ^{113}Cd spectrum of Hydrogel-544. The ^{113}Cd NMR spectrum of Hydrogel-544 shows two signals at 324.55 and 678.3 ppm, which correspond to CdS_2N_3O/$CdSN_3O_2$ and $Cd(S\text{-}GS)_4$ type coordination respectively[1].

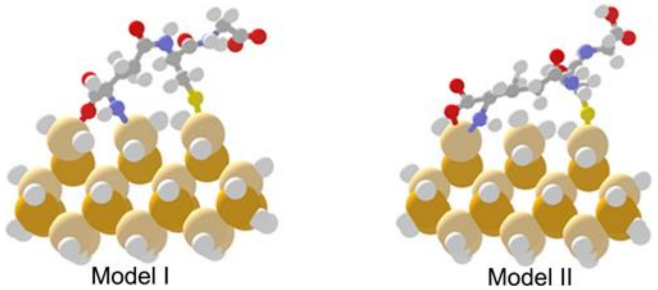

Supplementary Figure 14. Example of two binding models for the three-point bond (TPB) of GSH in CdTe NPs: Model I, where GSH binds to three different Cd atoms, and Model II, where GSH binds to two different Cd atoms. Our calculations show that Model I is preferred over Model II.

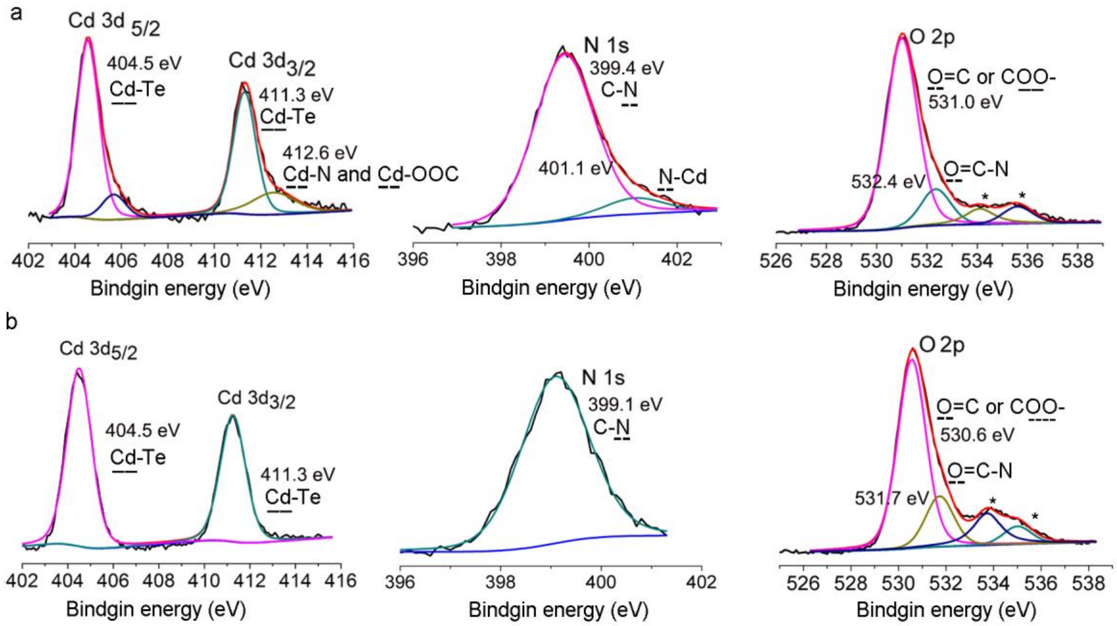

Supplementary Figure 15. XPS characterization of Hydrogel-544 (a) and Hydrogel-618 (b). The spectra fitted with Pseudo—Voigt (GL) functions with a combined polynomial and Shirley background. All fitted functions are based 20/80 ratio of Lorentzian-Gaussian. Full width at half maximum is adopted for the fitting functions as 1.3 eV for cadmium, 1.6 eV for nitrogen and 1.4 eV for oxygen. XPS were collected on a Kratos DLD Axis Ultra XPS using a monochromated Al source with energy resolution ~0.5 eV. The shoulder peaks marked by star in the O 2p energy level are from sodium contaminant in the NPs solution and water.

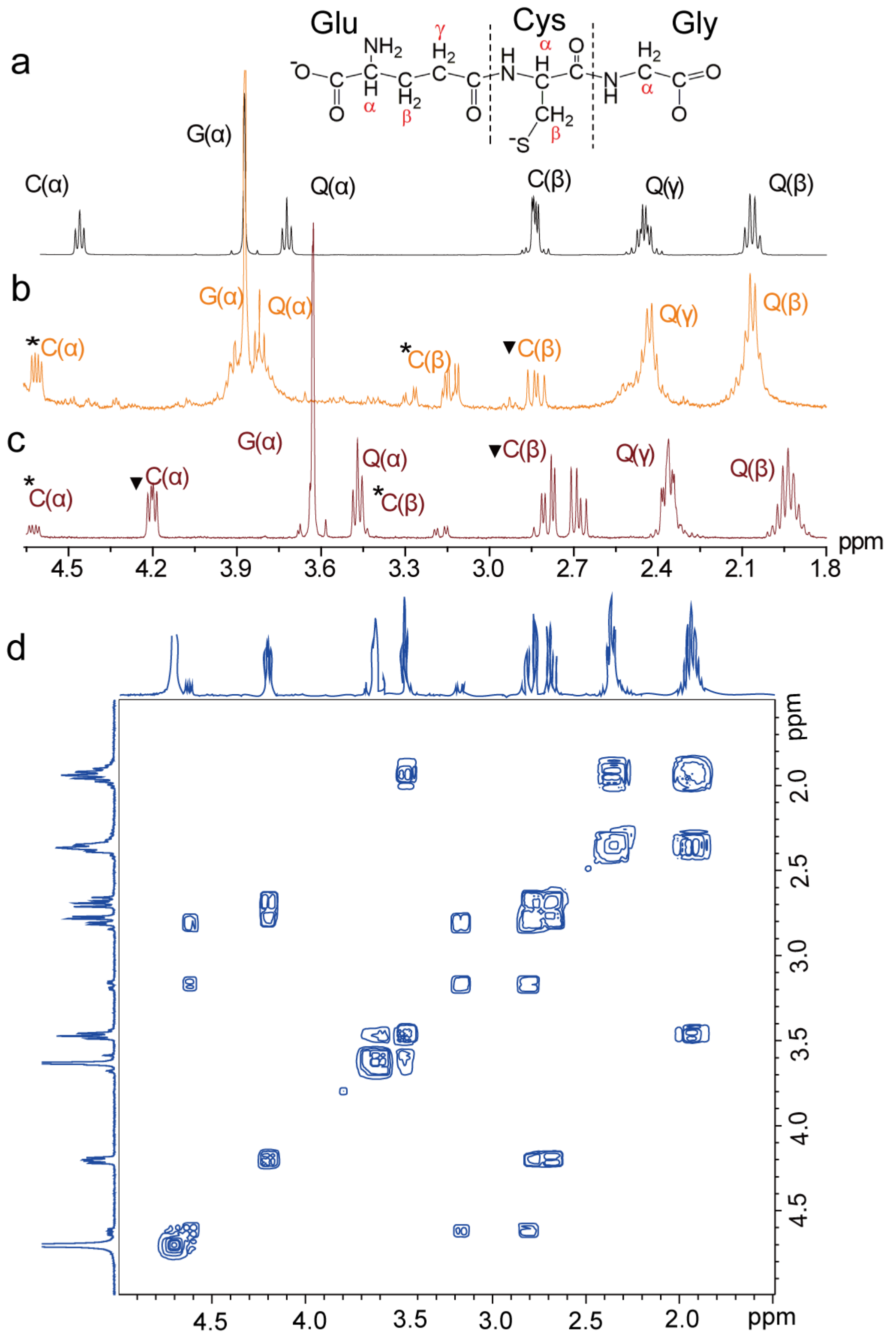

Supplementary Figure 16. Molecular structure of GSH stabilizers at the Au nanoparticle surface. Amino acid residues in GSH are indicated by a single letter code: Glutamyl, Q; Glycinyl, G; Cysteinyl, C. ^{1}H NMR spectra of (**a**) GSH at pH 3.5, (**b**) GSH-Au hydrogels, ∼3 nm (**c**) GSH-Au hydrogels, ∼8 nm. (**d**) Two-dimensional ^{1}H-^{1}H COSY spectrum of GSH-Au hydrogels, ∼8 nm. The two bonding modes are clearly visible in the NMR spectra (**b, c**) which ascribed to TPB (star) and SPB (triangle). ^{1}H-^{1}H COSY spectrum of GSH-Au 8 nm shows correlation within amino acid residues of TPB-GSH and SPB-GSH moieties. The ratio of TPB/SPB for 3 nm of GSH-Au hydrogel and 8 nm of GSH-Au hydrogels is 1:0.1 and 0.26:1 respectively.

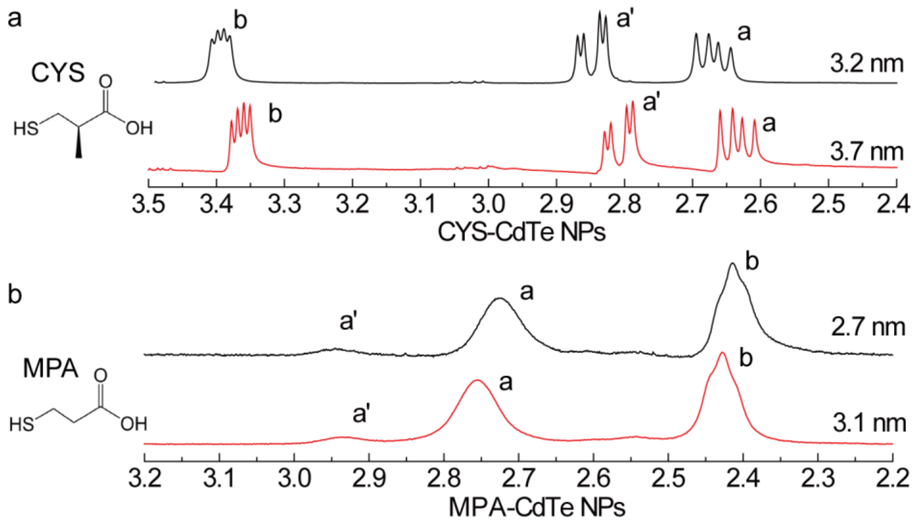

Supplementary Figure 17. One-dimensional ^{1}H spectrum of CYS-CdTe and MPA-CdTe NPs. Hydrogen atoms (a) in CYS and MPA split into two pairs of proton resonances (a and a′), which indicates the presence of two types of coordination geometries for CYS and MPA bond to the surface of NPs. The broad ^{1}H NMR signals are due to the compact packing MPA ligands inhibiting the molecule rotation.

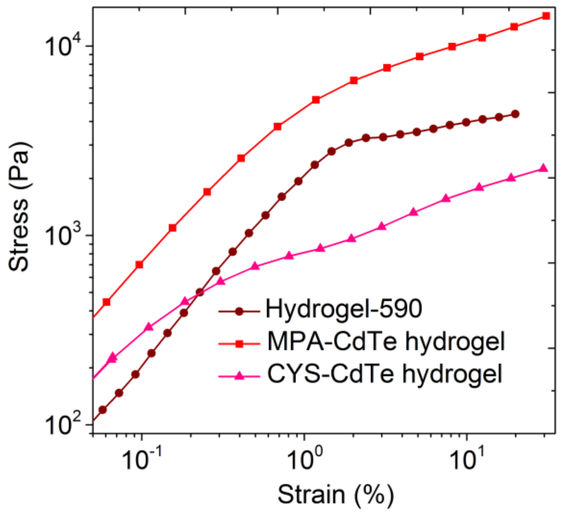

Supplementary Figure 18. Comparison of stiffness of Hydrogel-590, MPA-CdTe hydrogels and CYS-CdTe hydrogels. Hydrogel-590 (GSH-CdTe, CdTe core: 3.2 nm), MPA-CdTe hydrogels (CdTe core: 3.1 nm) and CYS-CdTe hydrogels (CdTe core: 3.2 nm).

Supplementary Table 4. The structure, major interaction forces, and viscoelasticity of typical hydrogels.

Hydrogel (Molecule weight, fraction)	Structure	Major interaction	Viscoelasticity G′ / G" in Pa	Reference
Hydrogel-544 (~38.4 KDa, 60%)	Porous NP solid	Hydrogen bond	$\sim 5\times10^{6}$ / $\sim 1.3\times10^{6}$	
Hydrogel-590 (~63.3 KDa, 58%)	Porous NP solid	Hydrogen bond	$\sim 1.2\times10^{4}$–1.9×10^{6} / $\sim 6\times10^{4}$–10^{6}	
Hydrogel-618 (~105.0 KDa, 54%)	Porous NP solid	Hydrogen bond	$\sim 5\times10^{3}$–1.3×10^{5} / $\sim 5\times10^{3}$–2×10^{5}	
GSH-Au gel (3 nm, 22 %w/w)	NA	NA	$\sim 3.2\times10^{4}/3.1\times10^{3}$	
MPA-CdTe gel (3.1 nm, 22% w/w)	NA	NA	$\sim 6.9\times10^{5}/1\times10^{5}$	
CYS-CdTe gel (3.2 nm, 22% w/w)	NA	NA	$\sim 2.1\times10^{6}/4.7\times10^{5}$	
Alumina (240 nm, 30 vol %)	Network structure	van der Waals force	$\sim 10^{2}$–10^{3} / $<10^{2}$	[2]
Silver NP-gel (NA)	Cross-linked fiber structure	Covalent bond	$<10^{5}$ / $<10^{4}$	[3]
CdSe NP gel (NA)	Network structure	Covalent bond	NA	[4]
PEG cross-linked actin gel (NA)	Cross-linked network structure	Covalent bond	$\sim 10^{3}$ / ~ 10	[5]
Fluid membrane-PEG2000 (6 %w/w, NA)	Lamellar	Covalent bond	10^{3}–10^{4} / 10^{2}–10^{3}	[6]
Collagen hydrogel (3.0×10^{5} g mol^{-1}, 35 mg/ml)	Cross-linked network structure	Hydrophobic and electrostatic attraction	$\sim 10^{3}$–10^{4} / 10^{2}–10^{4}	[7]
Poly(acrylamide) (1.6×10^{6} g mol^{-1}, NA)	Cross-linked network structure	Covalent bond	$\sim 10^{4}$–10^{6} / NA	[8]
Leucine zippers-polymers (20 KDa, 10%w/v)	Cross-linked network structure	Hydrophobic and electrostatic attraction	$\sim 10^{4}$ / $<10^{3}$	[9]
Peptide amphiphiles (1153 g mol^{-1}, 8.7 mM)	Cross-linked fiber structure	Hydrogen bond	~4500 / $<10^{3}$	[10]

*NA: not available

Supplementary References

[1] Mah, V. & Jalilehvand, F. Cadmium(II) complex formation with glutathione. J. Biol. Inorg. Chem. **15**, 441-458 (2010).

[2] Yanez, J. A., Laarz, E. & Bergström, L. Viscoelastic properties of particle gels. J. Colloid Inter. Sci. **209**, 162-172 (1999).

[3] Piepenbrock, M.-O. M., Clarke, N. & Steed, J. W. Rheology and silver nanoparticle templating in a bis(urea) silver metallogel. Soft Matter 7, 2412-2418 (2011).

[4] Pala, I. R., Arachchige, I. U., Georgiev, D. G. & Brock, S. L. Reversible gelation of II–VI nanocrystals: the nature of interparticle bonding and the origin of nanocrystal photochemical instability. Angew. Chem. Int. Ed. 49, 3661-3665 (2010).

[5] Sano, K.-I. et al. Self-repairing filamentous actin hydrogel with hierarchical structure. Biomacromolecules **12**, 4173-4177 (2011).

[6] Warriner, H. E., Idziak, S. H. J., Slack, N. L., Davidson, P. & Safinya, C. R. Lamellar biogels: Fluid-membrane-based hydrogels containing polymer lipids. Science **271**, 969-973 (1996).

[7] Rosenblatt, J., Devereux, B. & Wallace, D. G. Injectable collagen as a pH-sensitive hydrogel. Biomaterials **15**, 985-995 (1994).

[8] Muniz, E. C. & Geuskens, G. Compressive elastic modulus of polyacrylamide hydrogels and semi-IPNs with poly(N-isopropylacrylamide). Macromolecules **34**, 4480-4484 (2001).

[9] Liu, B., Lewis, A. K. & Shen, W. Physical hydrogels photo-cross-linked from self-assembled macromers for potential use in tissue engineering. Biomacromolecules **10**, 3182-3187 (2009).

[10] Greenfield, M. A., Hoffman, J. R., Olvera de la Cruz, M. & Stupp, S. I. Tunable mechanics of peptide nanofiber gels. Langmuir **26**, 3641-3647 (2009).
